# Supplementary figures and images for: SARS-CoV-2 sequencing artifacts associated with targeted PCR enrichment and read mapping
Source: PLoS One. 2025 Oct 16;20(10):e0334009. doi: 10.1371/journal.pone.0334009 (PMC12530606; doi:10.1371/journal.pone.0334009)

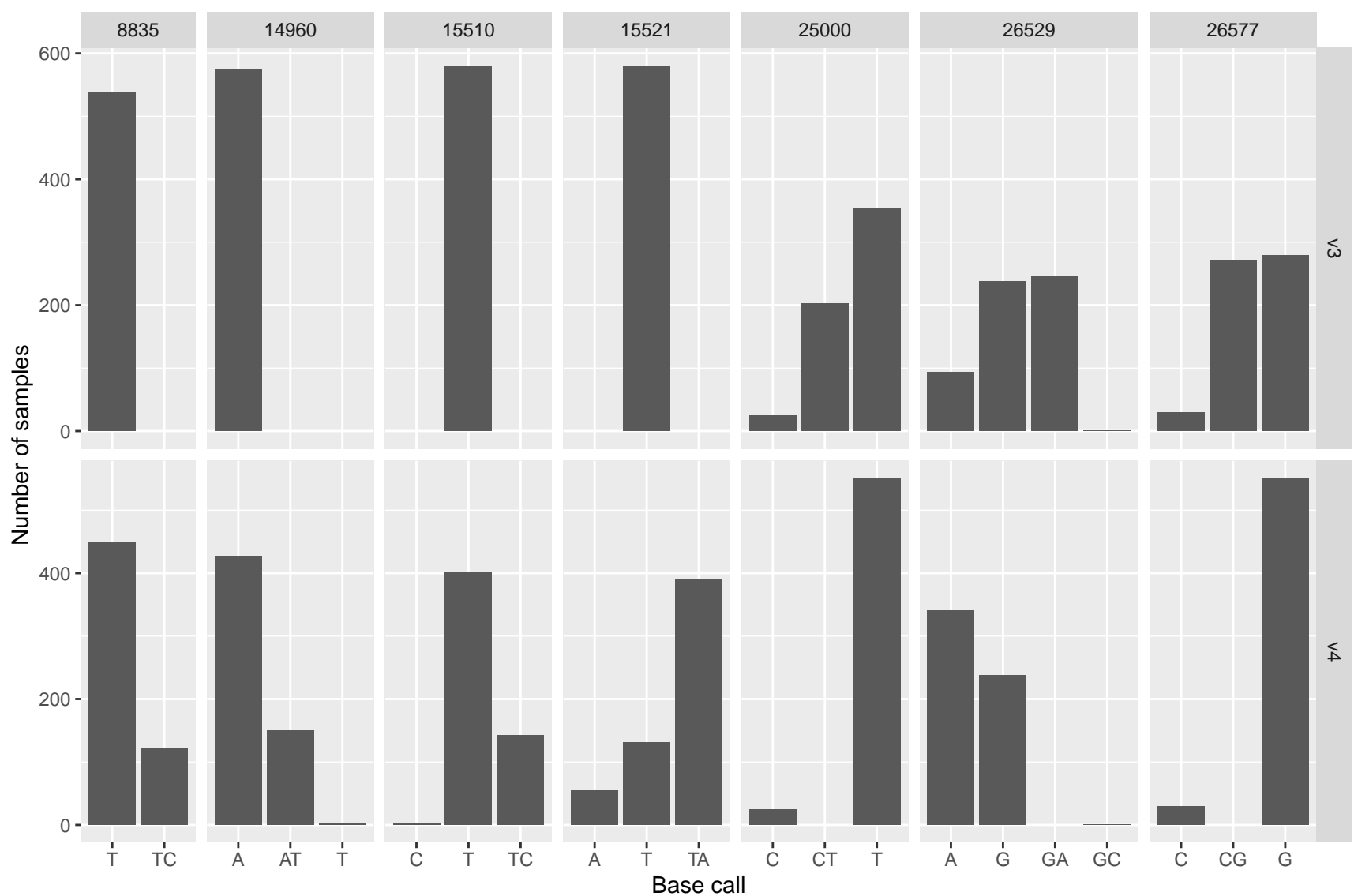

Supplement: S1 Fig — All base calls observed at each of the seven positions are shown on the x-axis, with the number of samples having the base call on the y-axis. (PDF) [file pone.0334009.s001.pdf]

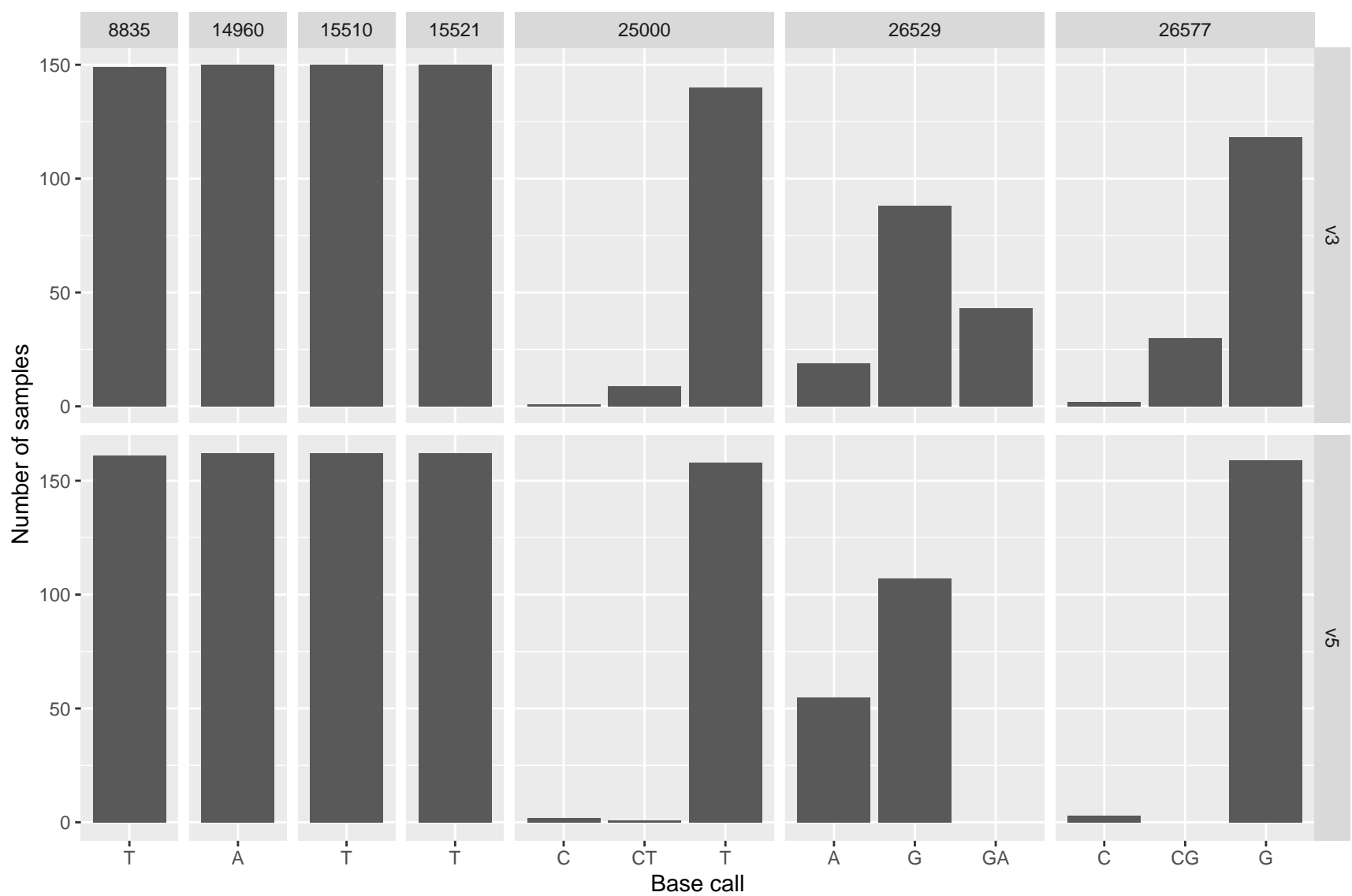

Supplement: S2 Fig — All base calls observed at each of the seven positions are shown on the x-axis, with the number of samples having the base call on the y-axis. (PDF) [file pone.0334009.s002.pdf]

Distribution of Artic V3 artifacts across sequencing plates

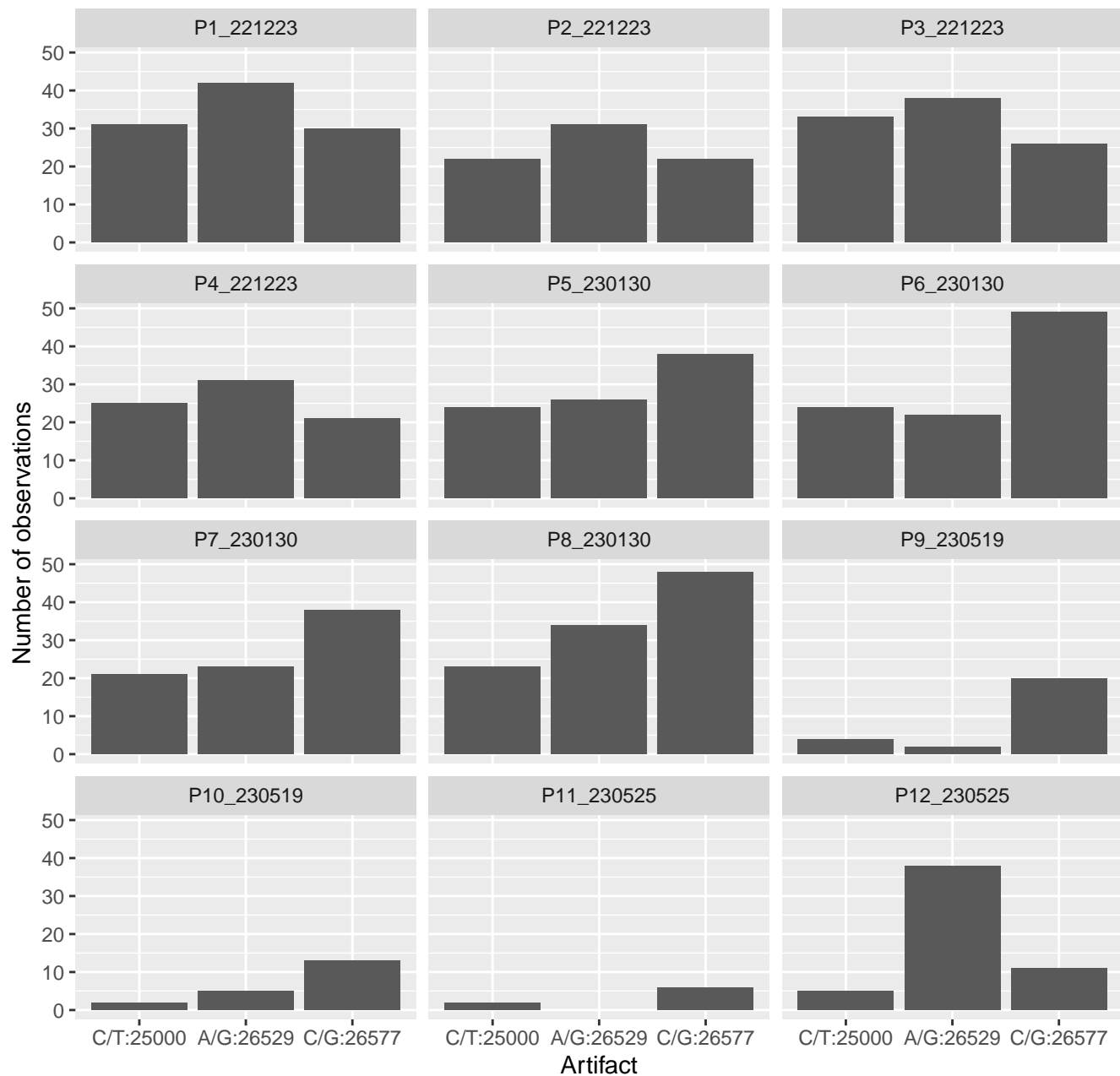

Supplement: S3 Fig — Each panel contains data for a plate of samples sequenced with two primer schemes, with the last six digits of the plate-id displaying the sequencing date. For each panel, the three positions where ambiguity is observed in Artic V3 with high frequency (see Table 2) are shown at the x-axis. The number of samples displaying inconsistency (ambiguous base call with Artic V3 and a clean base call with Artic V4.1/Artic V5.3.2) is shown on the y-axis. (PDF) [file pone.0334009.s003.pdf]

Distribution of Artic V4.1 artifacts across sequencing plates

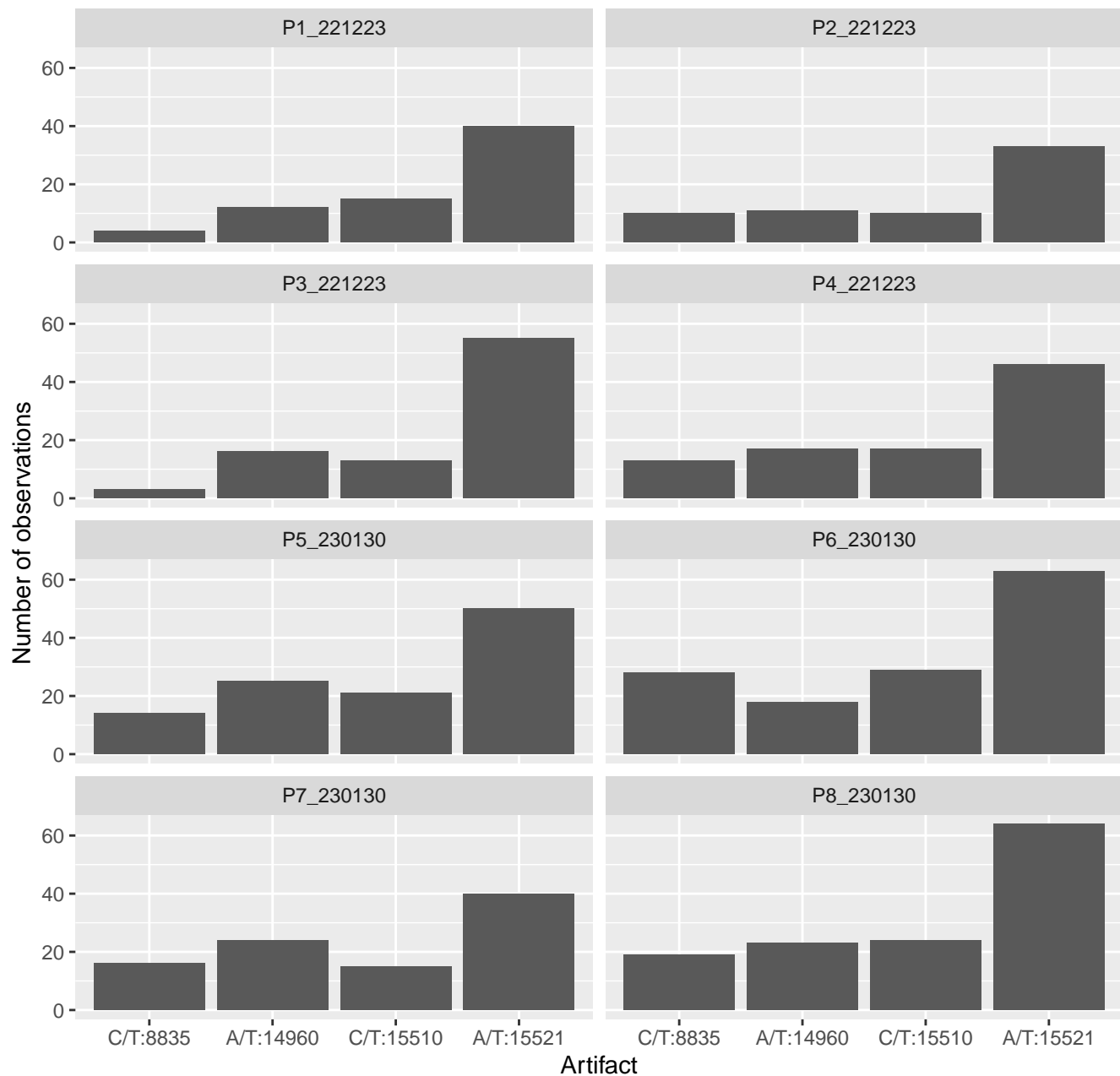

Supplement: S4 Fig — Each panel contains data for a plate of samples sequenced with both Artic V3 and Artic V4.1, with the last six digits of the plate-id displaying the sequencing date. For each panel, the four positions where ambiguity is observed in Artic V4.1 with high frequency (see Table 2) are shown at the x-axis. The number of samples displaying inconsistency (ambiguous base call with Artic V4.1 and a clean base call with Artic V3) is shown on the y-axis. (PDF) [file pone.0334009.s004.pdf]

# exp1: articV3

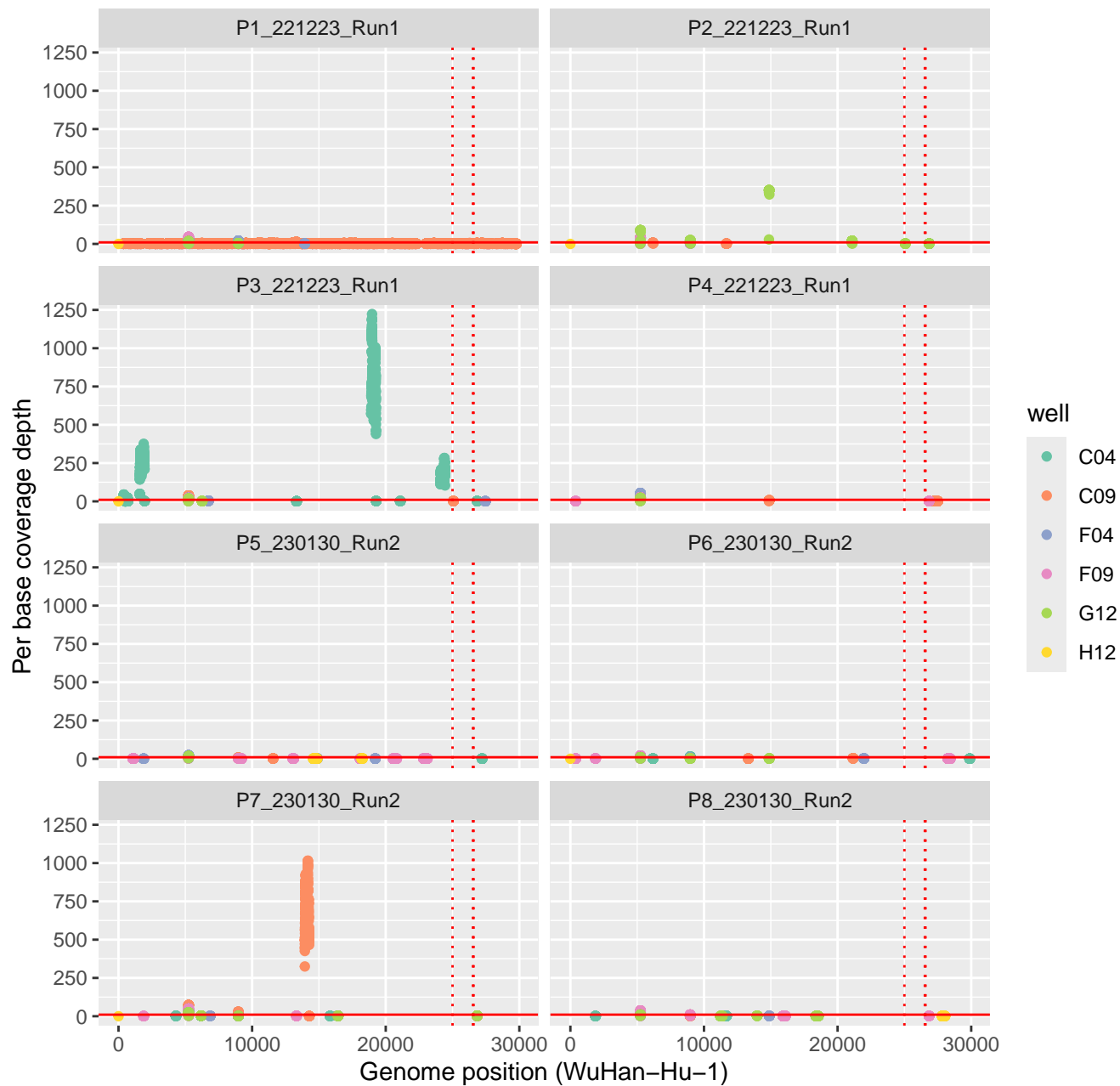

## exp1: articV4

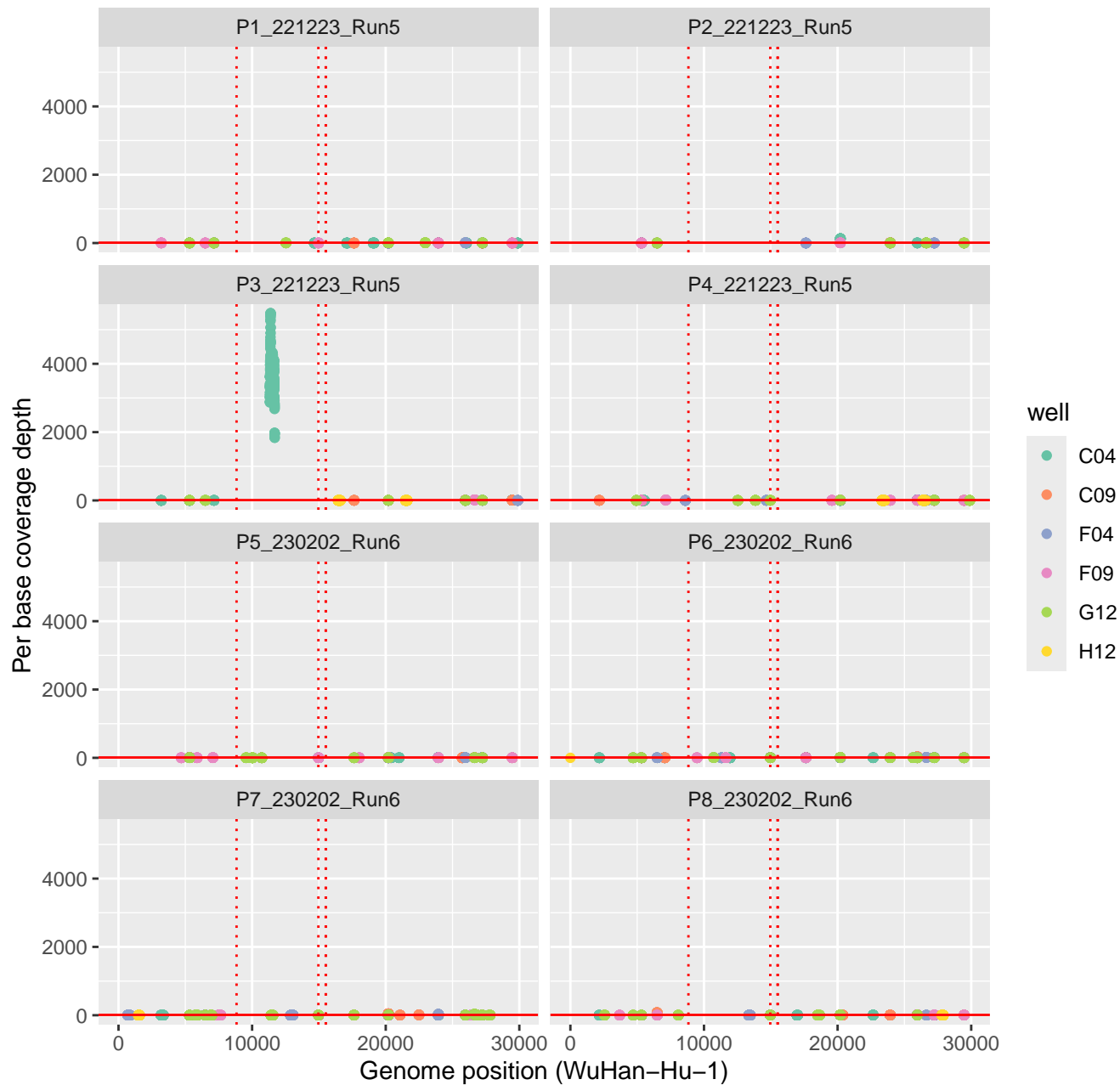

## exp2: articV3

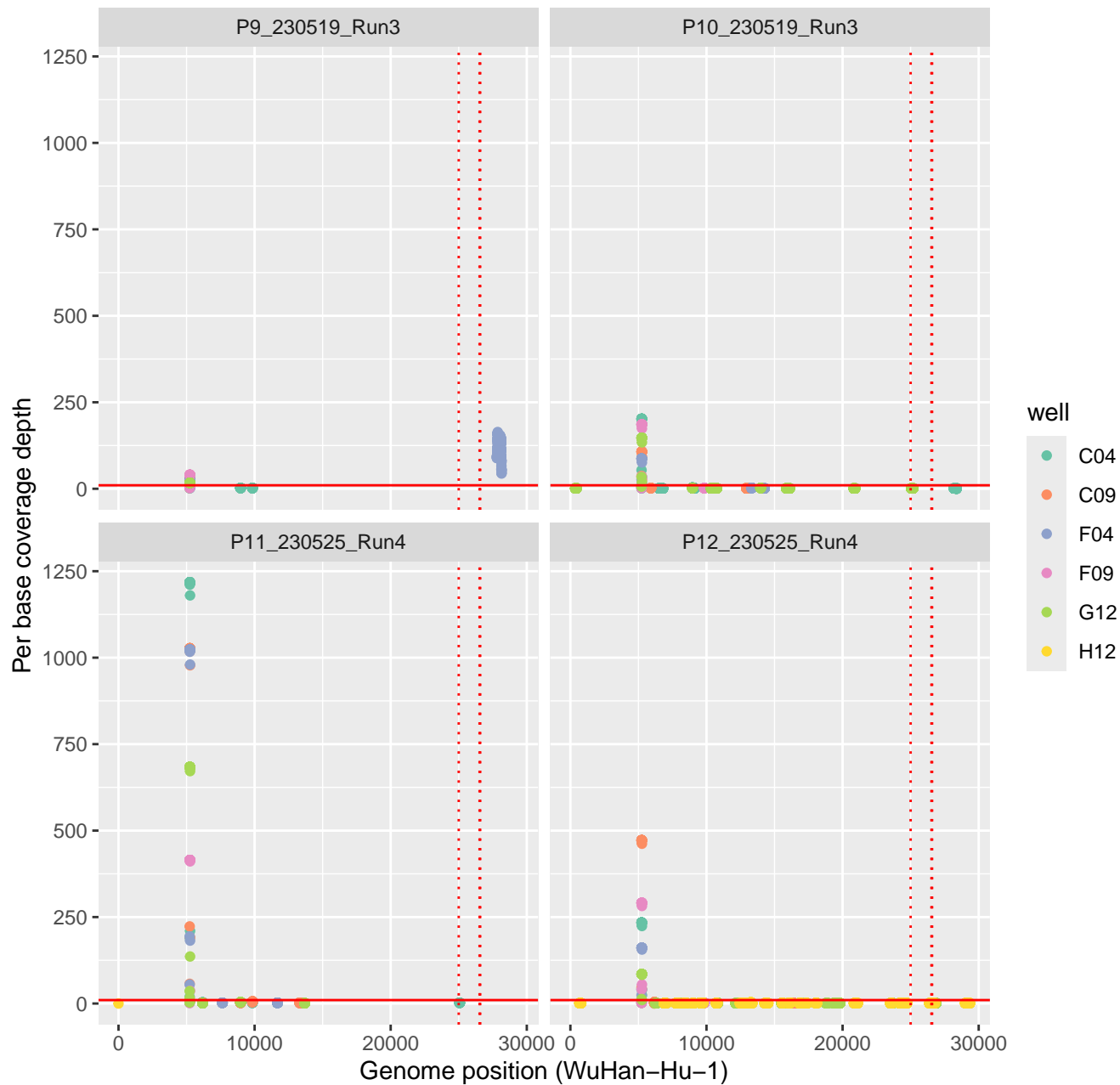

exp2: articV5

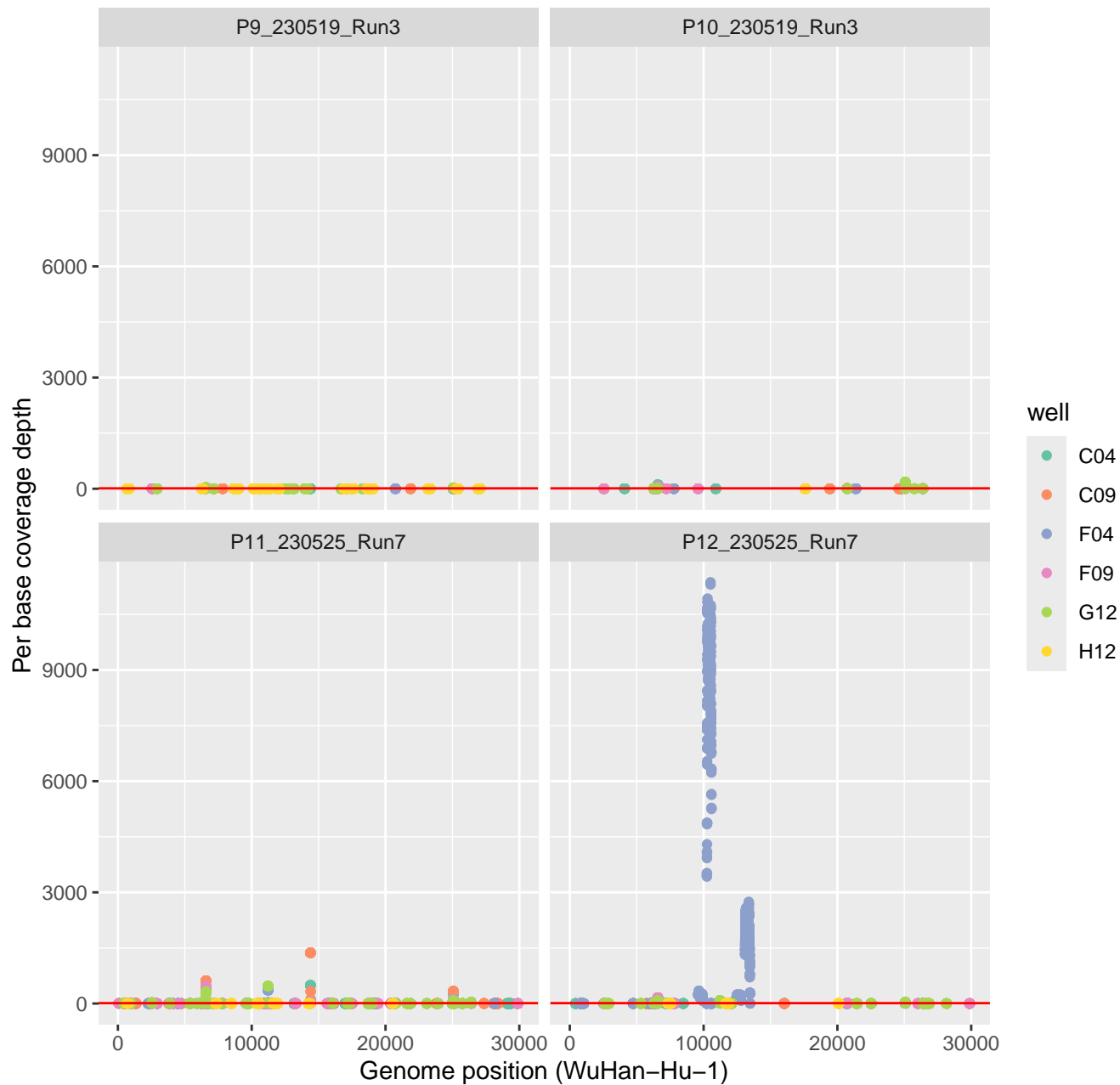

Supplement: S4 File — Mapped read coverage (depth) is shown for each of 24 sequencing plates used to generate the data in the “primer scheme” analysis (see Table 1). The titles of the plots denote the primer scheme used and experiment number (“exp1”: Artic V3 vs Artic V4.1, “exp2”: Artic V3 vs Artic V5.3.2). The plate-id for each sequencing plate consists of a plate-number (P1-P12, referring to a plate of samples sequenced with two primer schemes), the sequencing date, and a run-number (plates sequencing on the same run have the same run-id). Each plate was sequencing with a total of 6 negative controls (see Methods section), for which coverage is plotted in the same panel. Dotted lines in the panels show the positions where ambiguous base calls were observed with high frequency for the corresponding primer scheme. The red horizontal line denote the 10x coverage. (PDF) [file pone.0334009.s014.pdf]
