# Supplementary material for: SARS-CoV-2 sequencing artifacts associated with targeted PCR enrichment and read mapping
Source: PLoS One. 2025 Oct 16;20(10):e0334009. doi: 10.1371/journal.pone.0334009 (PMC12530606; doi:10.1371/journal.pone.0334009)
Supplement: S3 File — Screenshot 1 (page 1) shows alignments of reads supporting the minor allele at position 26,577 (of putative primer-origin), which were left untrimmed. Screenshot 2 (page 2) shows alignment of reads supporting substitutions only observed with the Artic V4.1 primer scheme. Screenshot 3−4 (pages 3−4) shows how reads from a sample of the SARS-CoV-2 variant “BA.2.86.x” get partially mapped in a 30 bp region of the spike-protein gene against the Wuhan-Hu-1 reference genome, while getting aligned inside the region when mapping against our internal BA2 consensus reference genome. A screenshot of the alignment of the three reference genomes analyzed in the current study is shown on page 5, where the differences responsible for the differential mapping in the region can be inspected. (PDF) [file pone.0334009.s013.pdf]

IGV-screenshot 1. Related to ”Primer scheme analysis”, Artic V3

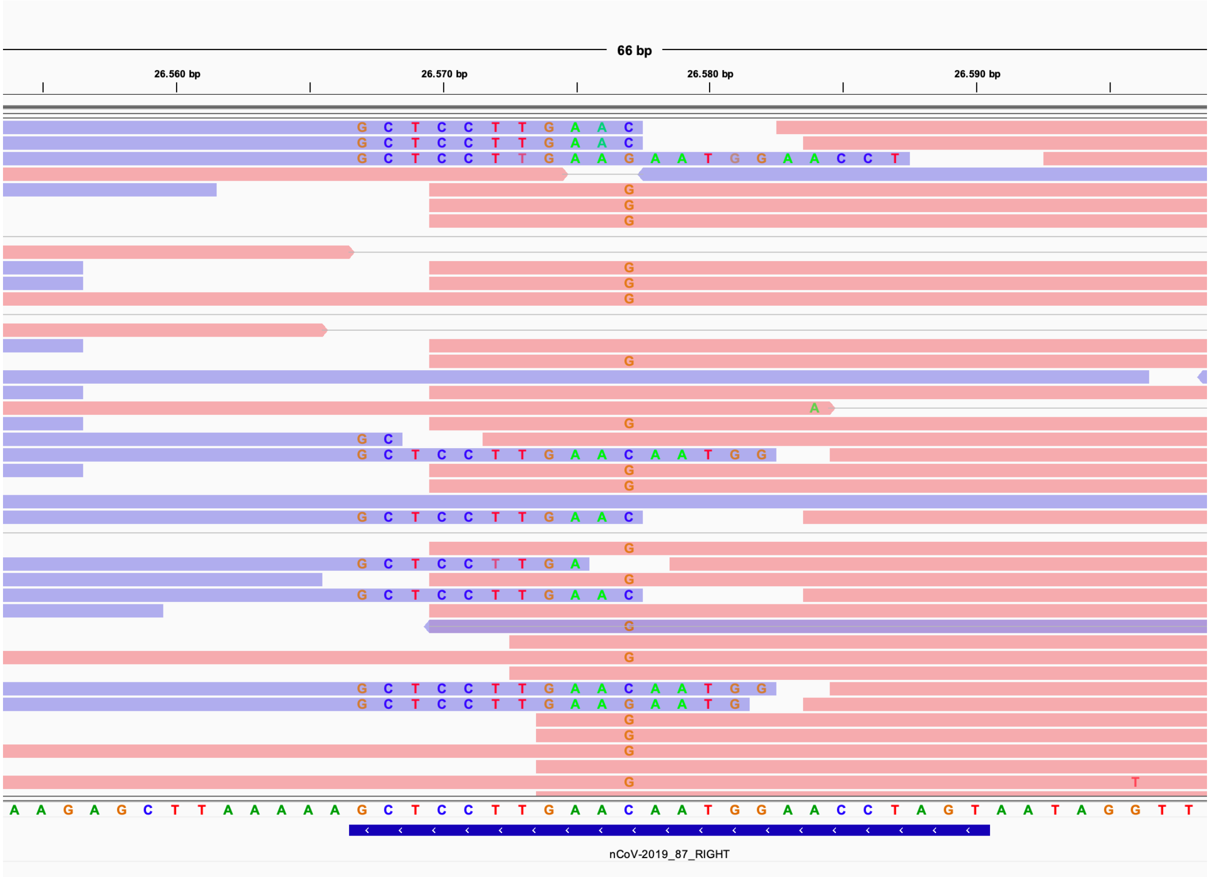

This screenshot was generated with a sample that had an ambiguous basecall with the artic V3 primer scheme at position 26,577. Reads mapping to the leading strand are shown in pink, reads mapping to the complement strand are shown in purple, and connections between reads of the same pair are shown with thin grey lines. The bases of the WuHan-Hu-1 reference genome are shown below the reads, as well as the artic V3 primer overlapping the position (87R). Bases within reads are shown for **both** mismatched bases and soft-clipped bases.

The longer stretches of bases visible within reads are soft-clipped bases generated by the primer-trimming software. They are all on reads mapping to the complement strand (in same direction as the primer), with alignments starting within the primer-region, consistent with the expected behaviour of the software.

Most reads mapping to the leading strand shows an SNV relative to the reference at position 26,577 (G). However, 7 reads in the screenshot have a C at the position instead (not highlighted, since its identical to the reference). These reads are not targeted by the primer trimming software, because they map on the leading strand. Likewise, one read mapping to the complement has a C at the position, and is left untrimmed because the alignment starts upstream of the primer. If the C comes from the primer, the reads must therefore derive from a PCR artifact (recombination or chimera formation during PCR amplification or library preparation).

IGV-screenshot 2. Related to ”Primer scheme analysis”, Artic V4.1

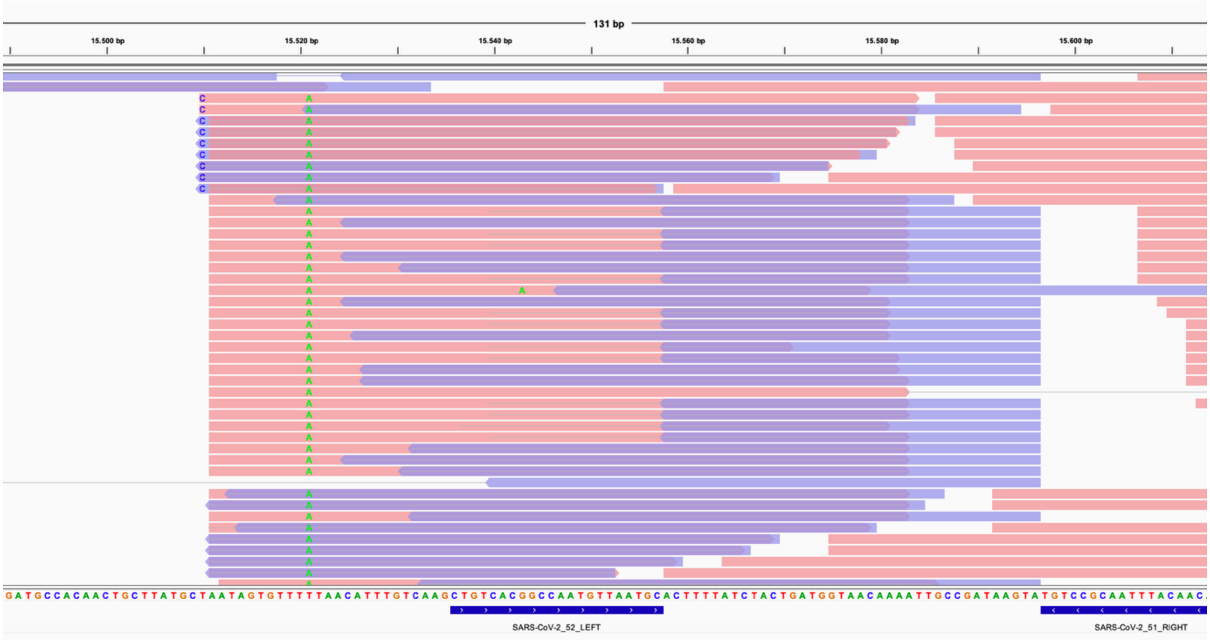

This screenshot was generated with a sample that had ambiguous basecalls with the artic V4.1 primer scheme at positions 15,510 and 15,521. Reads mapping to the leading strand are shown in pink, reads mapping to the complement strand are shown in purple, and connections between reads of the same pair are shown with thin grey lines. The bases of the Wuhan-Hu-1 reference genome are shown below the reads, as well as two of the primers in the region (52F, 51R). Bases within reads are shown **only** for mismatched bases.

The screenshot shows 9 reads supporting a C at position 15,510, and a large number of reads supporting an A at position 15,521. As these positions are not in a primer-region, they are not subject to primer-trimming. There are no other mismatches in the shown region relative to the reference genome, and the read alignments are therefore trivial.

In the absence of PCR artifacts, the reads supporting the two substitutions must come from amplicon 51, as they align upstream of the 52F primer region. However, if amplicon 51 was correctly amplified, there should be coverage across across the whole amplicon (as shown for all 17 samples in Fig.1, that is not the case). Therefore, the coverage profile is inconsistent with both amplicon 51 and 52, suggesting that reads are derived from a PCR artifact.

IGV-screenshot 3. Related to ”Reference genome analysis”, Wuhan-Hu-1 reference

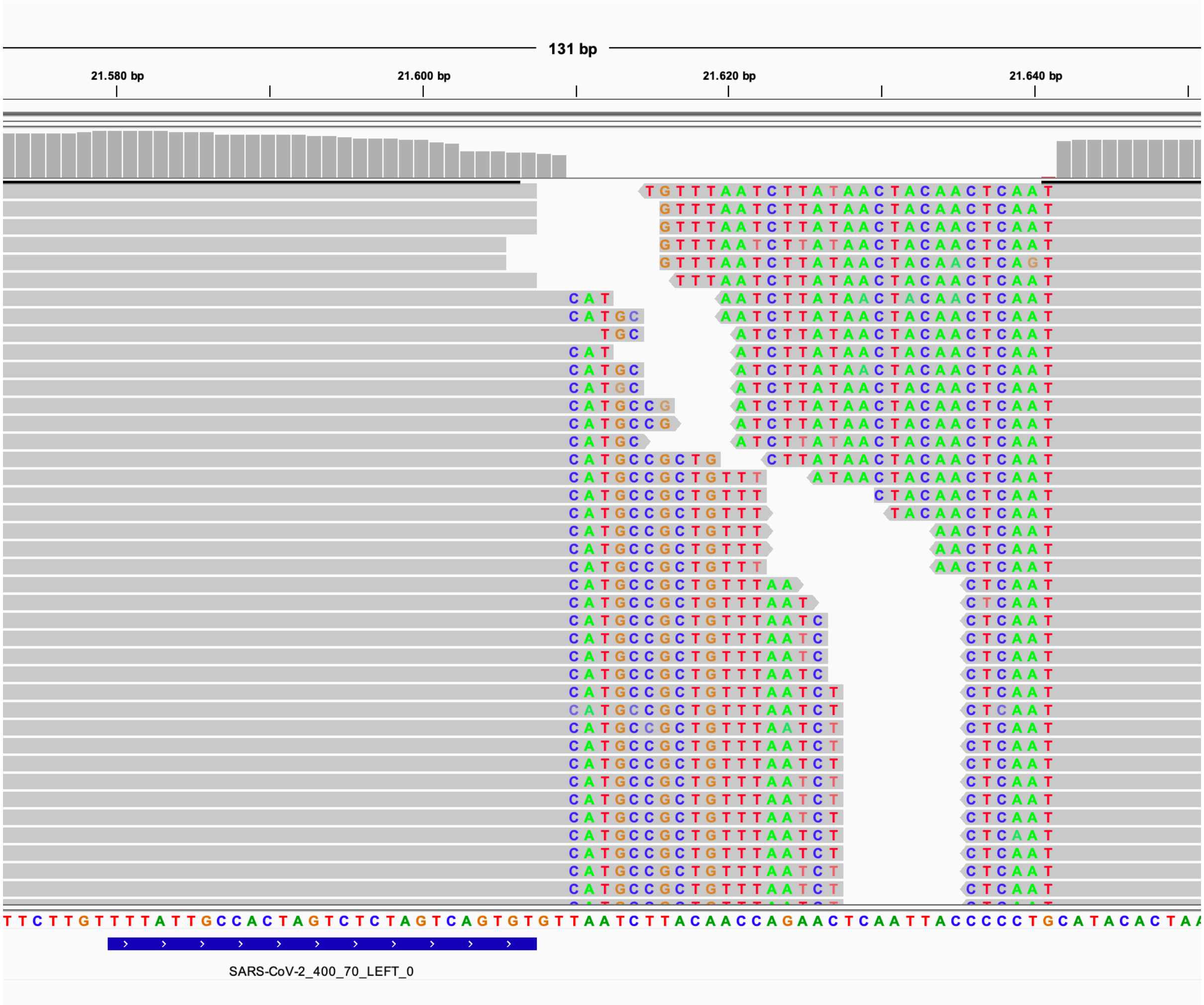

This screenshot was generated with a sample of the BA2.86.x variant, mapped against the Wuhan-Hu-1 reference genome. The bases of the reference genome are shown below the reads, as well as a primer of the Artic V5.3.2 primer-scheme (70L). Bases within reads are shown for **both** mismatched and soft-clipped bases.

The screenshot shows that the bases in the region from 21,610-21,640 get soft-clipped for all reads, regardless of the strand. These soft-clips are generated by the mapping software (BWA-MEM), because it cannot determine how to map the reads. The 12bp insertion in the region (21608:TCATGCCGCTGT) is visible in the soft-clipped reads.

IGV-screenshot 4. Related to "Reference genome analysis", BA2 consensus reference

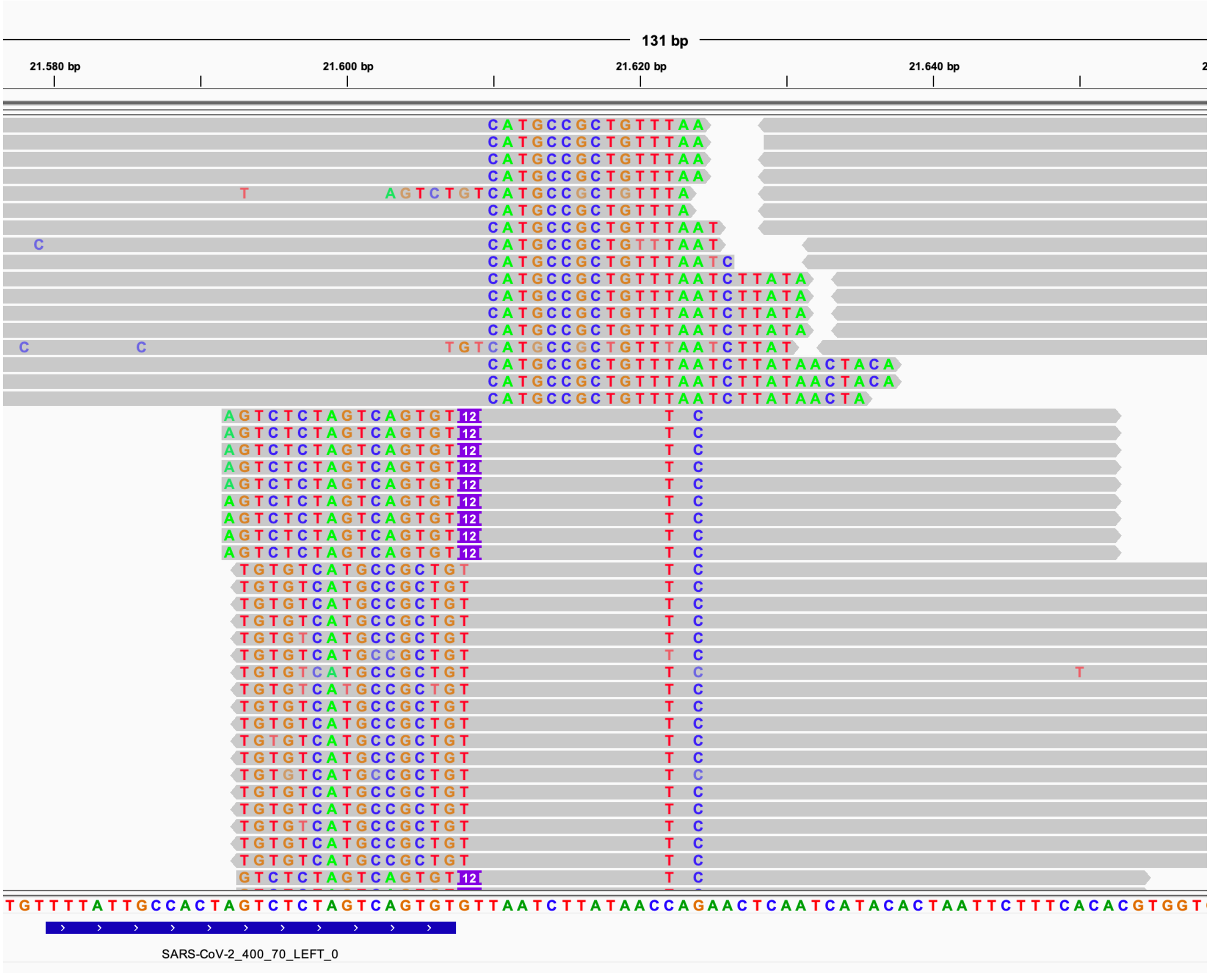

This screenshot was generated with a sample of the BA2.86.x variant, mapped against our internal BA2 consensus reference genome. The bases of the reference genome are shown below the reads, as well as a primer of the Artic V5.3.2 primer-scheme (70L). Bases within reads are shown for **both** mismatched and soft-clipped bases.

10 reads supporting the 12bp insertion (21608:TCATGCCGCTGT) are highlighted in purple with the number "12" by IGV. The sequences of these reads extend to both sides of the insertion (although they are soft-clipped upstream). The remaining reads are also consistent with the insertion, but have been soft-clipped at the ends by the mapping software because the alignment on one side of the insertion is too short.

Two SNVs (C21622T, G21624C) are also visible and supported by all reads that were not soft-clipped. Note, the SNV at position 21618 (C21618T) and the 9bp deletion (21633-21641) are not highlighted by IGV, because they are shared with the BA2 consensus reference genome. Thus, aside from the 12bp insertion, the mapping against this reference genome is straightforward.

Mafft screenshot: Related to ”Reference genome analysis”

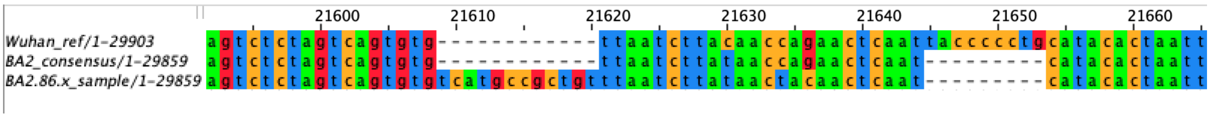

This screenshot shows a section of a genome alignment generated with mafft and visualized with Jalview.

The top sequence is the Wuhan-Hu-1 reference genome, the second sequence is our internal BA2 consensus reference genome, and the third sequence is a genome of the BA2.86.x variant (generated by mapping to the BA2 consensus reference genome).

The two reference genomes (Wuhan-Hu-1 and BA2 consensus) only differ by a single SNV and a 9bp indel (positions 21.630 and 21645-21653 in the alignment shown). It is therefore likely that the 9bp deletion (which is shared between the BA2 consensus reference genome and the BA2.86.x sample) is instrumental in providing an anchor for read mapping in the region.

Note that the positions shown refer to the specific alignment, which is different from the positions in the Wuhan-Hu-1 reference genome used when denoting SNVs and indels.
