# Supplementary material for: SARS-CoV-2 sequencing artifacts associated with targeted PCR enrichment and read mapping
Source: PLoS One. 2025 Oct 16;20(10):e0334009. doi: 10.1371/journal.pone.0334009 (PMC12530606; doi:10.1371/journal.pone.0334009)
Supplement: Attachment — Submitted filename: Rebuttal_250707.docx [file pone.0334009.s016.docx]

**Journal Requirements:**

1. Please ensure that your manuscript meets PLOS ONE's style requirements, including those for file naming. The PLOS ONE style templates can be found at

https://journals.plos.org/plosone/s/file?id=wjVg/PLOSOne_formatting_sample_main_body.pdf and 
<https://journals.plos.org/plosone/s/file?id=ba62/PLOSOne_formatting_sample_title_authors_affiliations.pdf>

We have checked that the manuscript meets the style requirements and that the files are correctly named.

2.  Please note that PLOS ONE has specific guidelines on code sharing for submissions in which author-generated code underpins the findings in the manuscript. In these cases, we expect all author-generated code to be made available without restrictions upon publication of the work.

Please review our guidelines at https://journals.plos.org/plosone/s/materials-and-software-sharing#loc-sharing-code and ensure that your code is shared in a way that follows best practice and facilitates reproducibility and reuse.

We have shared the code used to process the data in File S2, and have added a sentence to the data availability section, so the information is also included there.

3. We are unable to open your Supporting Information file [Table_S3 to Table_S5(.tsv)]. Please kindly revise as necessary and re-upload.

We have converted the files to excel-format.

4. We note that there is identifying data in the Supporting Information file <File_S3.csv>. Due to the inclusion of these potentially identifying data, we have removed this file from your file inventory. Prior to sharing human research participant data, authors should consult with an ethics committee to ensure data are shared in accordance with participant consent and all applicable local laws.

Data sharing should never compromise participant privacy. It is therefore not appropriate to publicly share personally identifiable data on human research participants. The following are examples of data that should not be shared:

-Name, initials, physical address
-Ages more specific than whole numbers
-Internet protocol (IP) address
-Specific dates (birth dates, death dates, examination dates, etc.)
-Contact information such as phone number or email address
-Location data
-ID numbers that seem specific (long numbers, include initials, titled “Hospital ID”) rather than random (small numbers in numerical order)

Data that are not directly identifying may also be inappropriate to share, as in combination they can become identifying. For example, data collected from a small group of participants, vulnerable populations, or private groups should not be shared if they involve indirect identifiers (such as sex, ethnicity, location, etc.) that may risk the identification of study participants.

Additional guidance on preparing raw data for publication can be found in our Data Policy (https://journals.plos.org/plosone/s/data-availability#loc-human-research-participant-data-and-other-sensitive-data) and in the following article: http://www.bmj.com/content/340/bmj.c181.long.

Please remove or anonymize all personal information, ensure that the data shared are in accordance with participant consent, and re-upload a fully anonymized data set. Please note that spreadsheet columns with personal information must be removed and not hidden as all hidden columns will appear in the published file.

File S3 did not contain any personal information. We included sampling week for the analysis “amb_over_time”, so Fig.3 could be reproduced (which is also fully anonymous, as we have ensured to use a minimum of 100 samples per data-point, with sampling location only specified as “Denmark”). However, as the data is not critical for the study, and most of the information is also available on ENA, we have removed the file from the submission.

**Reviewer #1 (ScreenIT Report)**

Flow charts and attrition. The experimental overview of the analyses and study design is shown in Table 1 in the manuscript.

Randomization. Not relevant for the experimental designs used in the current study.

Blinding. Not relevant for the experimental designs used in the current study.

Power analysis. Not relevant, the study is qualitative, and as such does not involve statstical analysis.

Software. We have added RRIDs for the tools which are currently in the repository, includingTrim Galore, BWA, iVaR, SAMtools, IGV, Rstudio, tidyverse, gridExtra, stringr, ggplot2, RcolorBrewer, mafft and Jalview.

Open code/data. We have provided the code used for processing the data in File S2, as a bash-script. Since it is very short (50 lines) and consists of software developed by others, we think the bash-script should be sufficient. The raw data has been made fully available on ENA, and we have also included result-files from Nextclade in the manuscript.

Self-acknowledged limitations. Limitations of the study are included in the manuscript text where relevant, fx. L376-379 (contamination could be responsible for artifacts observed at low frequency), L456-458 (limitation on interpretation due to lack of data generated without PCR enrichement), as well as in multiple places in the discussion.

Conflict of interst/Funding: We have filled out the form regarding Funding and competing interest during manuscript submission, and expect this information to be included in the publication.

**Reviewer #2:**

This article deals with some important questions concerning viral genome sequencing and in general the article is well written and coherent. The article focuses on how the choice of tools which are used in that process, both lab reagents and bioinformatic software, can impact the resulting consensus genomes. Due to the widespread adoption of these techniques, it is important to investigate these potential issues, and I commend the authors for highlighting concerns around primer choices. I also commend the authors for making all of their sequences available, as well as the code used in consensus sequence generation.

Thank you! We are very glad to hear that the reviewer finds the topic relevant and appreciate the work we put into making the code and raw sequence data fully available.

There have been plenty of examples of amplicon-based primer schemes producing spurious results due to unexpected primer binding, as well as the use of inappropriate bioinformatics tools which can lead to primers not being correctly removed from the resulting data, and this paper seeks to add to that knowledge base. Unfortunately, at the moment I do not believe that the authors have conclusively proven some of the points which they have set out to make, for reasons which I will outline below, but with some further work they should be able to do so.


Major Issues:

Firstly, the negative contamination criteria seems problematic to me in the context of this study (detailed in lines 217 to 232). From my understanding of it, the negative controls can have any number of mapped reads up to the 25th percentile, in which case some samples will be classed as a failure depending on their ct value. This seems overly permissive of contamination to me. With a median mapped read value of around 1.4 million for some samples (line 360), you could imagine a scenario where the negative control could have thousands of mapped reads and the whole run would not be considered contaminated. Furthermore, due to the variable sequencing depth achieved with amplicon methods, as the authors themselves have outlined in line ~436 of the discussion and line 328, the incorporation of unexpected amplicons can easily lead to spurious calls in regions of low coverage in a particular sample. This is further highlighted by the authors when they state that they increased their cut-off for ambiguous bases from 5 to 10 for this study (line 232), a metric they normally use to indicate contamination of the run.

We agree that the thresholds used for contamination QC are not very strict. The thresholds described are nevertheless the ones used throughout the pandemic at SSI, and we find it important to share them exactly as they were applied. Since the data was produced primarily for surveillance, there was a trade-off between the desired data quality and the risk of removing valuable data (or delaying it). For the purpose of surveillance, it is important that the data is released as quickly as possible and that new variants don’t go undetected due to strict QC-thresholds. Furthermore, due to the massive scale of sequencing, it was necessary to develop an automated filtering procedure, with fixed and consistent thresholds. However, most sequencing runs would have passed with a more strict threshold.

It is possible that contamination is not an issue with these samples, but the way the negative control filtering has been described and performed means that as a reader I feel I don’t have enough information to rule out the possibility that the results are due to contamination. This in turn diminishes the credibility for the findings in the “Impact of primer scheme on base calls” section. In my opinion almost all of the claims in this section could be due to contamination, especially as ambiguous base calls are a hallmark of run contamination.

To make the claims in this section sound, it is my opinion that the authors need a much stricter criteria for excluding samples based on potential negative contamination. A frequently used cut-off would be if there is more read depth for a given site in the negative control than the depth of the consensus caller (10 reads in this study (line 213)), the run would be considered contaminated and would not be taken forwards. The authors at least need to detail how many reads mapped and what the maximum depth of their negative controls were in order to satisfactorily rule out contamination as being the root cause of these mixed bases.

We agree with the reviewer that contamination can be a source of ambiguous basecalls and it is valuable to include data for the controls sequenced with the samples in the study. We have now included coverage information for all the controls used in the primer scheme analysis (S3 Fig, S3 Table), and we have added a paragraph with these results on L369-379. S3 Fig displays per base coverage (depth) for the controls along the Wuhan-Hu-1 reference genome. The coverage is generally below 10x across the genome (as highlighted by the red line), but is higher at specific positions (note the y-axis though, it is still very low levels of contamination here). S3 Table contains coverage information for all the negative controls for the positions where we observe recurrent ambiguous basecalls (as listed in Table 2). Most controls have no coverage at all at these positions, and the coverage depth is max 5x at all positions for all controls (S3 Table).

We agree that it is possible that some of the inconsistencies observed at low frequency could be caused by sample-to-sample contamination in a run-specific manner, and we have added this possibility to the text (L376). However, it is unlikely that contamination by chance should produce recurrent ambiguous base calls (affecting more than 10% of the samples) in a primer-scheme-specific manner.

There are additional arguments for why PCR artifacts are a more likely explanation than contamination:

1. **Primer scheme specifics for Artic V3**. The ambiguous basecalls observed when sequencing with Artic V3 (Table2: pos 25,000, 26,529, 26,577) are all in primer regions specifically for this primer scheme (primers 83L, 88L, 87R), and the minor alleles coincide with the sequence of the primers. This was not written in the manuscript text, we apologize for the omission. It is an important argument, and it has now been added on L311-313. Therefore, we know that the primers will generate ambiguity at these positions if not completely removed during processing (by primer trimming).
2. **Coverage profile inconsistencies for Artic V4.1.** Three of the ambiguous base-calls observed when sequencing with Artic V4.1 (Table2: pos 14,960, 15,510, 15,521) are in a region where the coverage profile is inconsistent with the primer scheme. The regions highlighted in red in Fig.1 are only covered by amplicons 49 and 51. However, if these two amplicons were correctly amplified, we should have coverage across the whole amplicons (as observed for amplicon 50 in Fig 1). We have edited the text to make it clear that the coverage of these amplicons indicates that they were not correctly amplified (L347-349).

Since the initial submission of the current manuscript, we have also become aware that some of the artifacts we have found relating to Artic V4 have been described previously, where they were linked to unintended primer binding in the region. We have added references to the preprint by Maio et al and the blogpost in which two of the PCR artifacts were initially described in the discussion (L476).

Secondly, the authors frequently suggest that mis-mapping of reads is the cause of some of the artefacts seen throughout the paper but don’t demonstrate this in any way. Mis-mapping reads can be seen if the bam files are inspected, or perhaps there would be an increase or decrease in the number of reads mapped at a given site. For example, lines 377-379, the authors claim that there is a difference in how the reads map in this region but show no evidence of this, and it could be easily viewed in (e.g.) IGV. In lines 381-384, the authors say that there is better mapping in this region but how is this measured?

We show how the choice of reference genome directly impacts read mapping in Fig 2 (which is also the most dramatic change we found), but it is correct that we have not provided similar plots for all observed inconsistencies.

The differences in the genome sequences described in lines 377-379 and 381-384 (in the submitted manuscript) are part of the reference genome analysis, in which the reference genome used is the only variable. The sequence data used to generate the genomes is otherwise identical, and the data was processed with the same software and settings, so it is hard to think of another explanation than read-mapping for the differences observed.

But we agree that it can be very informative to look directly at the read alignments, and we have now included two IGV screenshots detailing how the reads map to the Wuhan-Hu-1 and BA2 consensus reference genomes in an exemplary sample (pages 3-4, S3 File). We have also included a screenshot of the reference genome alignment (page 5, S3 File), which can be used to understand the underlying reasons for the differential mapping.

It seems to me that there is a reasonable chance that a proportion of these findings are issues with the bioinformatics tools used in the genome assembly, rather than an inherent primer scheme issue. For example, are the positions listed in line 297 all in primer binding sites?

Yes, the ambiguous basecalls listed on L297 are all in primer-binding regions specifically for artic V3, and we also know that the primer-sequences contain the minor allele. This information is indeed relevant and has now been included (L311-312). We thank the reviewer for bringing this omission to our attention.

If ambiguous calls are in primer sites is that an indication of incomplete primer trimming by iVar? The authors allude to the primer trimming software being the issue in line 433. It feels to me that this could be investigated further and could be the root cause of some of these artefacts rather than the primer scheme. It is a worthwhile finding to point out flaws in commonly used bioinformatics tools, but this is not investigated currently.

We have included an IGV screenshot for a sample with an ambiguous basecall in a primer-region (page 1, File S3), which provides further evidence for the notion that reads of primer origin were left untrimmed by the primer trimming software because they don’t follow the “rules” used by the software, due to PCR artifacts (as previously discussed). However, we think the data included in the current study is insufficient to make firm conclusions on this point.

We agree with the reviewer that an in-depth analysis of primer trimming software would be very interesting, but it would also be a major undertaking requiring much more data (ideally also targeted experimental data, quantifying PCR artifacts), so we think it is beyond the scope of the current manuscript. But we hope the manuscript will inspire others to follow up on this important topic.

Thirdly, with regards to figure 3, it seems to me that this graph ends too early. There is some increase in the ambiguous calls over time certainly, but the lineage prevalence is essentially the same for weeks 52,1,2,3,4, whereas the fraction of ambiguous bases doesn't really change until week 2, then dips, then is up for week 4. How do weeks 5+ look? If your rational is correct it will presumably continue increasing or plateau at this higher level? I think this figure needs to be expanded.

Unfortunately, the reason why the graph does not continue is simply that we did not have sufficient data. We set the minimum number of samples to 100 samples per week and the numbers decreased rapidly after peaking over Christmas/New-Year. We have added the detail about number of samples used to the methods section (L276).

Minor issues:

Line 45: That reference appears to not say that the virus is becoming less virulent – “A popular and incorrect view on the evolution of virulence, frequently expressed in the context of SARS-CoV-2, is that in the long run, pathogens will tend to evolve to be decreasingly virulent” (<https://www.nature.com/articles/s41579-023-00878-2>)

We have replaced the reference to one by Carabelli *et al*., which has a paragraph on the relative severity of SARS-CoV-2 variants, and have also modified the text to reflect that multiple factors are involved:

*“Five years later, SARS-CoV-2 has evolved to become more transmissible but also less severe, in part due to increased population immunity, and in response the global sequencing effort has been scaled down”* (L43).

line 294: How many were left after this criteria?

Table 2 contains all positions found according to the cut-off, with the dataset used. We have modified the text to:

*“A total of seven positions generated recurrent inconsistent base calls for more than 10% of the samples in the two primer scheme comparisons (Table 2)”* (L307) to make it clear that the data in table 2 is not a selection, but the full result.

line 307: I don't understand how there is a range of % here.

The range refers to the different values for each of the four positions with inconsistent base calls when sequencing with Artic V4.1. We have edited the text as follows to make the sentence more understandable:

*“Furthermore, the dominant base for the ambiguous base calls generated with Artic V4.1 was inconsistent with the base calls generated with Artic V3 and V5.3.1 in a large fraction of the samples (ranging from 14% of the samples at position 8835 to 52% of the samples at position 15,521”)* (L337).

line 325: Can the authors investigate what mechanism would generate this artefact? Could a different primer be binding to that region? Or is the library prep fragmentation process favouring that area?

As also described further up, it turns out that some of the artefacts we found associated with Artic V4.1 have been described previously (we were unaware about this at the time the manuscript was submitted). The inconsistent coverage is indeed (at least in part) caused by unintended primer binding in the region. We now refer to the preprint and original blogpost in the discussion (L476).

line 328: This is the exact scenario where a permissive definition of contamination in the negative control can lead to mixed/erroneous base calls.

Agreed. We have addressed the possible issue with contamination further up. Also note that the alternative bases called only when sequencing with Artic V4.1 are not observed any of the samples when sequencing with Artic V3 (nor in any of the samples sequenced with Artic V3/Artic V5.3.2), as shown in S1-2 Figs.

line 395: Samples sequenced in the author’s lab?

Yes, all samples analysed in the current study (outlined in Table 1) were sequenced at SSI, as part of the routine SARS-CoV-2 surveillance, including the 8865 samples used in the “Ambiguous base calls over time” analysis, to which L.395 belongs.

line 430: Where the snp falls in a given read can be investigated by looking at the alignments and would go some way towards answering this question.

Agreed, we have included an IGV screenshot for one of the positions generating an ambiguous basecall in a primer region with Artic V3 (page 1, S3 File S3), providing further support for the notion that reads go untrimmed due to PCR artifacts.
