# Supplementary material for: SARS-CoV-2 sequencing artifacts associated with targeted PCR enrichment and read mapping
Source: PLoS One. 2025 Oct 16;20(10):e0334009. doi: 10.1371/journal.pone.0334009 (PMC12530606; doi:10.1371/journal.pone.0334009)
Supplement: Attachment — Submitted filename: Rebuttal_250901.docx [file pone.0334009.s017.docx]

From reviewer:

”I thank the authors for the efforts they have made towards resolving the concerns I highlighted in the first round of review. I think that the work has made positive steps forward and a lot of my issues have been resolved. However, I still have concerns regarding the base calling inconsistencies that the authors have detailed.”

I disagree that the contamination shown is only minor. Having positions with over 9000x coverage clearly shows that you have significant levels of negative control contamination (exp2: articV5). Your choice of dots for the plot have made it difficult to judge the exact size of the areas of contamination but presumably they are in the region of a 100 bases+. I also disagree with the assertion that “it is unlikely that contamination by chance should produce recurrent ambiguous base calls (affecting more than 10% of the samples) in a primer-scheme-specific manner.” Recurrent low-level ambiguous base calls would be exactly the sort of thing that would be visible when contamination occurs across a plate. I reject the assertion that this was a necessary sacrifice for surveillance sequencing purposes. For example the COG-UK consortium sequencing teams worked to a standard where if there was more than 100 mapped reads in a negative, the run would be considered contaminated (https://www.medrxiv.org/content/10.1101/2021.10.09.21264695v1.full; <https://pmc.ncbi.nlm.nih.gov/articles/PMC8461472/>).

More importantly though, as the authors themselves have stated these SNPs are all in primer binding sites so this is really an issue with primer trimming software. I agree that the SNPs are potentially caused by poor primer trimming, but then that isn’t a primer scheme issue per se it is a bioinformatics issue and as such should be an issue on the iVar github page. However, the SNP at position 26577 is on the leading strand at that site (as shown in S3 file) and is due to not having a read be trimmed. There shouldn’t be an amplicon produced as there isn’t a forward primer expected to bind there so a trimming sorftware would not look at those reads? Can the authors suggest beyond that it is a primer trimming artefact what is going on for these sites? What is it about V4.1 that is causing this?

If these SNPs are real PCR artefacts beyond an issue in the lab, the authors should be able to show that these SNP artefacts can be seen across the enormous selection of SARS-CoV-2 sequences sequenced with the ARTIC primers that are out there. Clearly the SNPs that others have identified can be assumed to be larger issues, but what about the SNPs that are new to this study, can they be seen in samples further afield? Presumably they would appear as low frequency variants elsewhere too.

In summary, I think the authors need to demonstrate that these SNPs are an issue that affects others outside of their lab by showing they are present in SARS-CoV-2 genomes on a public repository, given that their sequencing run shows signs of contamination.

We appreciate the time and effort the reviewer has put into the review, and are glad to hear that the reviewer also appreciates that we have done our best to address all concerns raised in the first review. We will address the remaining concerns in a topic-wise pattern here below.

**Contamination in routine surveillance**

The question of what constitutes an appropriate threshold of contamination for routine surveillance is not, in our opinion, the immediate focus of the current study, but given the reviewers comments we would nevertheless like to address a few of them.

For the first study the reviewer refers to (Alikhan *et al.*), they write:

*A sequencing run was considered as reliable when: the associated negative controls for that sequencing run had less than 100 total SARS-CoV-2 mapped reads; less than 4% genome recovery (equating to 3 amplicons); and the sequencing run itself encountered no issues impacting the quality of the read data*.

but there is no information of the number of negative controls used, their positions on the plate, or the protocol step at which they were introduced. For the second study (Page *et al.*), they write:

*Samples were prepared and sequenced in 96-well plates with one cDNA-negative control per plate and one RNA extraction-negative control, where applicable.*

At SSI (and in the current study), we use 6 negative controls on every 96-well plate, without exception, 4 of which are used throughout the protocol (including RNA extraction, where the risk of contamination is highest). We typically see contamination in just one of these 4 main controls, indicative of sample-to-sample contamination (not reagent contamination). The chance of detecting such contamination is obviously much higher if you include more controls.

Regarding the number of mapped reads in negative controls, this number is strongly influenced by what is considered "mapped", as well as the overall sequencing depth. In the first study mentioned, they filtered their mapped illumina reads to a minimum alignment length of 148bp (for 150bp reads), to get rid of background mapping from for example primer-dimers. We have deliberately not applied any filters on the mapped reads. If we had used such a strict alignment threshold, we could probably reduce the number of mapped reads with at least 50% (thus removing a lot more than just primer-dimers). We have chosen to use multiple controls and no filtering, to specifically maximize our ability to detect different types of contamination.

**Could the artifacts we report in Table 2 be the result of recurrent contamination?**

It is correct that there are specific positions with high coverage in the negative controls, but they represent a very minor fraction of the genome. We apologize if this was not clear from the plots we provided, we have now calculated the genome recovery for the 144 negative controls used, the average is 0.2%. We have also updated the plots of contamination, to make the distribution across plates and controls clearer (S4 File). In S4 File, coverage is now shown separately for each sequencing plate, and we have labelled them so that plates sequenced in the same run or on the same date can easily be identified.

From these updated plots, it should be clear that the contamination is restricted and localized. For example, there is evidence of contamination at a specific region on plate ”P7_230130_Run2” (sequenced with Artic V3), but this contamination is not present in any other controls on the same plate, nor on any other plates in the run. Thus, this is a case of localized sample-to-sample contamination (not reagent contamination). This type of contamination can never be ruled out, even with a lot of negative controls, therefore our analysis is focused on inconsistencies occurring with very high frequency.

We have added two supplementary figures (S3-4 Figs), which further highlights how prevalent the inconsistencies are, as well as their distribution across all sequencing plates. We agree with the reviewer that contamination can be recurrent, but if a contamination had occurred affecting so many samples on all sequencing plates, we would expect to find evidence of it in at least some of the negative controls used on the plates. To summarise the data reported in Table S3 further:

For Artic V3:

- A total of 7 mapped reads were found across 72 negative controls at positions 25,000, 26,529 and 26,577

For Artic V4:

- No mapped read coverage at positions 8835, 15,510 and 15,521 in any of the 48 negative controls used, a total of 38 reads mapped to position 14,960 (distributed across 15/48 controls)

Furthermore, none of the 7 reads mapping the positions reported for Artic V3 support the minor base (responsible for the ambiguity). Thus, except for position 14,960, we found no evidence of the inconsistencies reported in Table 2 in any of the negative controls used with the primer schemes.

We have added these results to the manuscript (L.368-389), and hope that the reviewer will agree with our assessment; That, given the number of replicates and controls used, contamination is unlikely to be responsible for the artifacts reported in Table 2.

**If not contamination, then what?**

The minor base, generating ambiguity on the three positions reported for Artic V3 in table 2, is contained in the primer-mix of Artic V3 (and only Artic V3). However, we do not think this is an issue with the primer trimming software. iVar should only trim reads if they align in the same direction as the primers, and the alignment starts in the primer-region. This is not the case for the reads generating ambiguity, at least not in the samples we have investigated. iVar has handled the reads in the sample shown in File S3 (p1) correctly. We therefore conclude that the ambiguity-causing reads are generated in the lab, during PCR. If chimeras are formed, combining DNA from primers with target DNA, we would expect to see reads like the ones shown in FileS3, p1.

Chimera formation during PCR is a very well documented artifact, which has been known for more than 30 years (<https://doi.org/10.1093/nar/18.7.1687>), and a large number of studies have been done on its impact and procedures to reduce it (see fx

<https://doi.org/10.1016/j.gene.2010.08.009>, https://doi.org/10.1093/nar/30.9.2083). The conditions for chimera formation are ideal in our case, where we have near-identical templates (differing by just one base) in the PCR-mix.

Still, as we do not provide any experimental evidence for chimera formation in the current study, we only mention it as a possibility. We have added a few more sentences and references on the topic (L. 487-490) to assist readers who may not be familiar with the phenomenon.

**Confirmation of reported artifacts from other labs**

We agree that it would strengthen our study to have confirmation of the reported artifacts from other studies. We have this confirmation for the artifacts we report associated with Artic V4 (thereby also demonstrating that our experimental design can pick up real artifacts). Unfortunately, it would be difficult to get confirmation for Artic V3, for the following reasons:

Artic V4 was rolled out in June 2021, and V4.1 in December 2021. However, at SSI, we decided to continue with Artic V3 (with added spike-in primers), which was an unusual decision. By September 2022 (first time-point of the samples analysed in the current study), most of the world had long since stopped using Artic V3. The timing is important, because the artifacts depend on the target genomes having a different base than the primers. Samples sequenced with Artic V3 in 2021 are not useful here.

The artifacts we observe with artic V3 are ambiguous base calls, not SNPs. They are therefore only visible in the genome sequence if the bioinformatic pipeline used reports ambiguity and will additionally depend on the ambiguity threshold. It is very common to simply report the dominant base, in which case this ambiguity will be hidden in any released genome sequence. We would therefore, most likely, have to collect raw data to detect it. While there is a lot of raw data available in public repositories like ENA, the primer-scheme is very rarely reported, because the submission form does not contain a dedicated field for it.

For these reasons, it is therefore, unfortunately, far from trivial to obtain external corroboration for the primer scheme artifacts we report as associated with Artic V3.

**Revision of the current manuscript**

With the current manuscript, we have added a lot more analyses and details on the distribution of contamination. We hope that these details have put the reviewer at ease regarding the contamination observed and its possible impact on the results we report.

We think that the manuscript is now completely transparent in regard to contamination, the report on the extent and distribution of contamination is very detailed. We have also updated our concluding sentence in the results section for the primer scheme analysis to:

*“Given that these base calling inconsistencies were observed with very high frequency across all the sequencing plates (S3-4 Figs), we would expect to see the inconsistencies in at least some of the negative controls, if they were the result of recurrent contamination.” (L.386)*

And we also mention the possibility of contamination in the discussion:

*“In the current study, we identified recurrent ambiguous base calls in multiple primer regions, despite having implemented primer trimming in our pipeline.* *While ambiguous base calls can also be the result of recurrent contamination, we found no evidence of such a contamination in the negative controls used (72 negative controls, distributed on 12 sequencing plates).” (L.480)*

With these sentences, we do not rule out contamination completely, just that our negative controls (which we have a lot of) do not support it.

Likewise, we do not rule out that there could be issues with the primer trimming software, just that we haven’t found evidence for it in the current study:

*“Inspection of the aligned reads did not uncover any issues with the primer trimming software either, as the reads generating ambiguity were mapped in the opposite direction of the primer or upstream of the primer region (page 1 in File S3).” (L.484)*

The possibility that the artifacts associated with Artic V3 is due to chimeras is mentioned as a possibility, not a proven fact:

*“Thus, if the reads originate from the primers, they are likely chimeric sequences formed by recombination during PCR (10). Chimeras are known to arise from incomplete primer extension during PCR, where partially extended products act as primers on heterologous templates (10,35,36). By having primers with bases distinct from the template in the PCR reaction, such a heterologous template is effectively generated.” (L.486)*

We hope that the reviewer will also be content with this solution.
